# Faster Detection of Poliomyelitis Outbreaks to Support Polio Eradication

**DOI:** 10.3201/eid2203.151394

**Published:** 2016-03

**Authors:** Isobel M. Blake, Paul Chenoweth, Hiro Okayasu, Christl A. Donnelly, R. Bruce Aylward, Nicholas C. Grassly

**Affiliations:** Imperial College London, London, UK (I.M. Blake, C.A. Donnelly, N.C. Grassly);; World Health Organization, Geneva, Switzerland (P. Chenoweth, H. Okayasu, R.B. Aylward)

**Keywords:** poliomyelitis, outbreak detection, spatiotemporal scan statistic, integrated nested Laplace approximation, INLA, spatiotemporal regression, eradication, viruses, polio, acute flaccid paralysis, response activities

## Abstract

As the global eradication of poliomyelitis approaches the final stages, prompt detection of new outbreaks is critical to enable a fast and effective outbreak response. Surveillance relies on reporting of acute flaccid paralysis (AFP) cases and laboratory confirmation through isolation of poliovirus from stool. However, delayed sample collection and testing can delay outbreak detection. We investigated whether weekly testing for clusters of AFP by location and time, using the Kulldorff scan statistic, could provide an early warning for outbreaks in 20 countries. A mixed-effects regression model was used to predict background rates of nonpolio AFP at the district level. In Tajikistan and Congo, testing for AFP clusters would have resulted in an outbreak warning 39 and 11 days, respectively, before official confirmation of large outbreaks. This method has relatively high specificity and could be integrated into the current polio information system to support rapid outbreak response activities.

## Abstract 2

Identification of spatiotemporal clustering of acute flaccid paralysis cases can accelerate outbreak detection and thereby support rapid response activities.

The global eradication of polio is entering its final stages. The last case of poliomyelitis associated with serotype 2 wild poliovirus was reported in 1999 and of serotype 3 in 2012. In Africa, the last reported case of serotype 1 wild poliovirus was in Somalia in August 2014. Transmission of this serotype has yet to be interrupted in Afghanistan and Pakistan, and in 2014, 359 serotype 1–associated cases were reported worldwide, 81% of which occurred in Pakistan (*1*).

Transmission of wild poliovirus persists in countries where the disease is endemic, but outbreaks can also occur in previously polio-free populations in which population immunity is not sustained. For example, the 2013 polio outbreak in the Middle East was linked to importation of poliovirus from Pakistan (*2*). The live-attenuated oral poliovirus vaccine (OPV) has played a huge role in achieving >99% reduction in global annual incidence of poliomyelitis, but its continued use also means there is a risk for emergence and spread of circulating vaccine-derived poliovirus (cVDPV) (*3*). In 2015, cVDPV outbreaks were reported in at least 5 countries (*1*). The risk for serotype 2 cVDPV may be heightened during the planned global switch from trivalent to bivalent (containing Sabin virus types 1 and 3) OPV during routine vaccination in April 2016 (*4*). Poliomyelitis outbreaks substantially raise the cost of the eradication program and hinder progress toward eradication, particularly if they are not swiftly controlled (*5*). Early detection is therefore critical to the program to enable a fast outbreak response to quickly stop transmission.

Surveillance for poliomyelitis relies on the reporting of cases of acute flaccid paralysis (AFP) in children <15 years of age by healthcare providers (Figure 1 at http://dx.doi.org/10.5281/zenodo.44361) (*4*,*6*). In some areas this surveillance is supplemented by environmental surveillance, which involves the periodic collection and testing of sewage samples for the presence of polioviruses. Surveillance is challenging because of the large number of asymptomatic cases (100–1,000 infections/AFP case) and because there are multiple causes of AFP (e.g., trauma, toxins, enteroviruses), thus requiring laboratory testing of stool samples to confirm the presence of poliovirus (*7*–*9*). 

**Figure 1:**
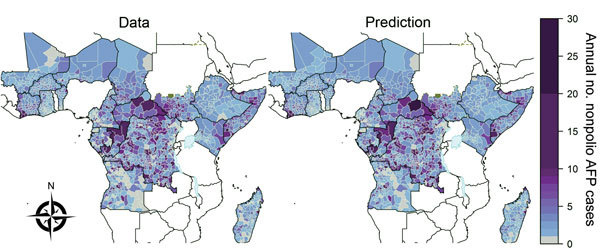
Nonpolio acute flaccid paralysis (AFP) cases in sub-Saharan Africa, 2003–2013. Left, mean annual number of cases reported at the second administrative unit (district) in countries in sub-Saharan Africa that have recently experienced a polio importation or outbreak or are considered to be at high risk for these events. Right, expected annual number of nonpolio acute flaccid paralysis cases reported at the district level; the number was obtained by fitting a spatiotemporal mixed-effects regression model to nonpolio AFP data from 2003–2013. Areas that report >25 annual cases are grouped into the 25–30 category (the maximum observed annual reported number was 128 in Tshopo, Democratic Republic of the Congo, in 2007). South Sudan gained independence in 2011, but reporting in this area before independence is shown for comparison. The publication of these maps does not imply the expression of any opinion whatsoever on the part of the World Health Organization (WHO) concerning the legal status of any territory, city, or area or of its authorities or concerning the delimitation of its frontiers or boundaries. WHO does not endorse or approve the use of subnational boundaries in this map. Disputed borders and areas are shown in green and lakes at borders are shown in pale blue.

In 2010, large outbreaks of poliomyelitis in Tajikistan and Republic of the Congo (Congo) were detected relatively late, partly due to delays in laboratory processing of stool samples; the delayed detection resulted in a limited effect from the outbreak response vaccination campaigns (*10*). The high transmissibility and pathogenicity of wild and vaccine-derived polioviruses means that poliomyelitis cases may be expected to cluster in space and time to a greater extent than do cases of AFP associated with other enteroviruses or noninfectious causes. We therefore decided to investigate whether clusters of AFP could herald poliomyelitis outbreaks and be identified as an early warning of outbreaks before laboratory confirmation.

## Methods

### Data

Cases of AFP are reported through a network of healthcare providers as part of routine surveillance for poliomyelitis (*6*). We analyzed 67,218 AFP cases with clinical onset during 2003–2013; the patients resided in 3 countries that had >150 confirmed cases of polio annually since 2005 (Tajikistan, Congo, and Somalia) or in countries in Africa considered to be at high risk for an outbreak of wild poliovirus. For each AFP case, the following information was recorded: the first and second administrative levels (province and district, respectively) in which the patient resided; the dates of AFP onset, case notification, and stool sample collection; and the patient’s age and sex. 

AFP cases with stool samples adequate for testing were distinguished as virologically confirmed cases of poliomyelitis caused by wild poliovirus type 1 or 3 (3,089 cases), cVDPV (70 cases), or nonpolio AFP. Cases of AFP in persons without adequate stool samples were defined as polio-compatible cases (i.e., cases with clinical symptoms compatible with poliomyelitis, as determined by a panel of experts; 1,436 cases) or nonpolio AFP cases. The total number of nonpolio AFP cases was 62,623.

Institutional ethics approval for this study was not sought because the databases are free of personally identifiable information. National and subnational (first administrative and second administrative levels, respectively) boundaries were obtained from the World Health Organization.

**Figure 2:**
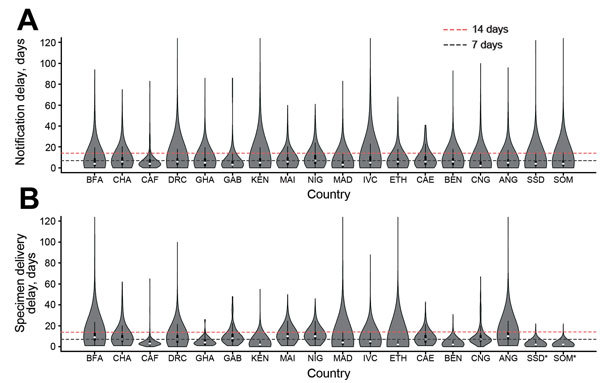
Distribution (violin plots) of time delays in notification of acute flaccid paralysis (AFP) cases and in sending samples for laboratory testing, by country, Africa, 2010–2013. A) Delay between onset of acute flaccid paralysis and notification of cases. B) Delay between notification of acute flaccid paralysis cases and the date collected stool samples were sent to a global polio laboratory. Asterisks (*) indicate that the date stool samples were sent to the laboratory was not available; in these instances, the date of the second stool collection was used instead. In the violin plots, white dots correspond to the median value, the rectangle indicates the interquartile range, and the vertical line corresponds to the range between upper and lower adjacent values. ANG, Angola; BEN, Benin; BFA, Burkina Faso; CAE, Cameroon; CAF, Central African Republic; CHA, Chad; CNG, Republic of the Congo; DRC, Democratic Republic of the Congo; ETH, Ethiopia; GAB, Gabon; GHA, Ghana; IVC, Côte D’Ivoire; KEN, Kenya; MAD, Madagascar; NIG, Niger; SOM, Somalia; SSD, South Sudan.

**Figure 3:**
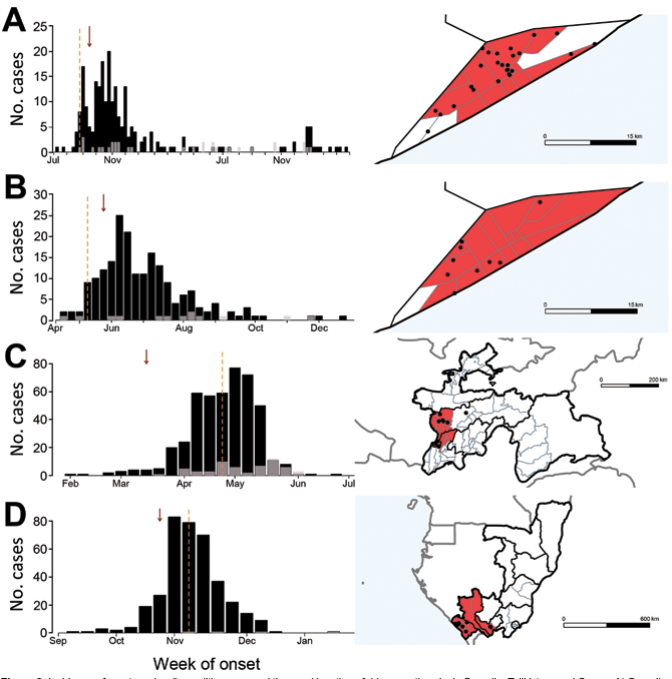
Incidence of serotype 1 poliomyelitis cases and time and location of 4 large outbreaks in Somalia, Tajikistan, and Congo: A) Somalia, 2005–2007; B) Somalia, 2013; C) Tajikistan, 2010; D) Congo, 2010. The charts on the left indicate weekly incidence of confirmed polio (black) and polio-compatible (gray) cases (by onset date of acute flaccid paralysis, AFP); vertical dashed lines indicate the date the outbreak was officially confirmed and arrows the date that an alarm would have been raised for detection of AFP clustering. Maps on the right show the second administrative divisions (districts) containing the significant cluster of acute flaccid paralysis cases; divisions are colored red if the alarm cylinder included its centroid. Each dot corresponds to a confirmed poliomyelitis case, plotted randomly within the corresponding district of occurrence. Maps in panels A and B show the administrative divisions of Banadir in Somalia. Gray lines in maps in panels C and D indicate neighboring countries. Blue shading indicates the sea. The publication of these maps does not imply the expression of any opinion whatsoever on the part of World Health Organization (WHO) concerning the legal status of any territory, city, or area or of its authorities or concerning the delimitation of its frontiers or boundaries. WHO does not endorse or approve the use of subnational boundaries in this map.

For each of the 20 countries in the study, we obtained raster population size data for 2010 from the WorldPop project (http://www.worldpop.org.uk) (*11*,*12*). The data contained estimates of population distribution at ≈100-m^2^ spatial resolution. Population size estimates at the district level were acquired by aggregating the raster data within each district by using the R package raster (*13*) (Figures 2 and 3 at http://dx.doi.org/10.5281/zenodo.44361) implemented in the R programming language (*14*).

### Time from Paralysis Onset to Case Notification to Specimen Delivery for Laboratory Testing

Delays in reporting and testing were determined for all AFP cases reported from Africa during 2010–2013 with available date information. We computed the length of time between paralysis onset and case notification and between case notification and the date stool samples were sent to a global polio laboratory for testing.

### Space–Time Analysis of Nonpolio AFP data

We fitted a mixed-effects spatiotemporal statistical model to the data for each country (http://dx.doi.org/10.5281/zenodo.44361). In brief, the number of nonpolio AFP cases reported in a district at a given time was assumed to follow a Poisson or negative binomial distribution. In accordance with the model of Besag, York, and Mollié (*15*), the linear predictor was based on spatially structured and spatially unstructured random effects, with an additional offset of population size, and a random walk over time to account for temporal trends in reporting. The models were fitted to the nonpolio AFP data for each country in a Bayesian framework by using INLA (integrated nested Laplace approximation) (*16*) implemented in the INLA package (*17*). We selected the most parsimonious model, according to the deviance information criterion (*18*), to determine whether the count data followed a Poisson or negative binomial distribution. 

### Creation of Real-Time AFP Databases

A record is not kept of when each AFP case enters the AFP database. To test whether clusters of AFP cases could be identified in advance of an outbreak, we created real-time AFP databases for each Monday during 2003–2013 by assuming cases entered the database on the date the case was notified by local healthcare providers. These real-time databases partly capture the delay between symptom onset and reporting of AFP cases (Figure 1 at http://dx.doi.org/10.5281/zenodo.44361) and are a best-case scenario of timely reporting. When the date of notification was missing, we used the date of investigation, first stool collection, or second stool collection (in that preferential order) as proxy for when the case entered the database.

### Testing of Real-Time AFP Databases to Detect Polio Outbreaks

For each country, the prospective Kulldorff Poisson space–time scan statistic (*19*) was evaluated at weekly intervals from the real-time AFP database to identify clustering of AFP cases in space and time. In summary, for every district in a given country, space–time cylinders were created; the cylinders were centered on the centroid of the district, and each had a different radius (representing various distances from the centroid to other district centroids) and height (representing different time periods up to and including the current week of surveillance). Cases of AFP were included in a given sized cylinder if the onset date for the case was within the interval of the start and end dates of the cylinder and the radius passed through the centroid of their reporting district. The cylinder end date was always the date of the real-time database; the start date varied from 1 to 90 days before the end date (we assumed standard methods of poliomyelitis outbreak detection would have detected an outbreak >90 days after the date of paralysis onset of the first AFP case). The maximum radius of the cylinder was restricted to 500 km, a conservative distance given the observed spatial clustering of polio cases at the start of an outbreak (Figure 9 at http://dx.doi.org/10.5281/zenodo.44361). The radius did not extend outside a given country. 

The number of AFP cases observed within each cylinder was summed, and the likelihood ratio function, defining how likely there is an elevated risk within the cylinder compared with outside the cylinder, was maximized across cylinders of all locations and sizes. The expected rate of AFP reporting in the absence of a polio outbreak was obtained from the spatiotemporal regression model. The cylinder with the maximum likelihood ratio corresponded to the identified cluster (http://dx.doi.org/10.5281/zenodo.44361). The p value of the cluster was determined by Monte Carlo hypothesis testing by simulating cases under the null hypothesis and comparing the rank of the maximum likelihood ratio of the data with the simulations (http://dx.doi.org/10.5281/zenodo.44361). A cluster of AFP cases was defined to trigger an alarm of a potential outbreak when p<0.05. The space–time scan statistic was evaluated by using SaTScan version 9.3 (*20*), which was called using the R programming language (*14*), and the computation was parallelized over a 16-core, high performance cluster. We also tested the ability of the space–time permutation scan statistic (*21*) as an alternative to the space–time Poisson scan statistic (http://dx.doi.org/10.5281/zenodo.44361) because the space–time permutation scan statistic only relies on case data.

An outbreak was classified as detected by the algorithm if a warning alarm was raised within the outbreak period and if the location of the alarm occurred in at least 1 district containing reported outbreak-associated polio cases. The algorithm was assessed in its ability to detect confirmed serotype 1 and 3 wild poliovirus and cVDPV outbreaks. An outbreak period was defined as the length of time that consecutive, type-specific cases occurred with dates of paralysis onset <6 months apart. The percentage of outbreaks that were correctly identified was recorded (sensitivity of algorithm). The time of the alarm was compared with the date the outbreak was officially confirmed. The date of confirmation was not available for the smaller outbreaks; therefore, it was not possible to evaluate the timeliness of these alarms, apart from observing the time between the alarm and the date of onset of the first case. The specificity of the cluster detection algorithm was evaluated at the country level as the percentage of outbreak-free weeks without a false alarm. Sensitivity to this definition was examined (http://dx.doi.org/10.5281/zenodo.44361).

## Results

### Nonpolio AFP Reporting

The number of nonpolio AFP cases reported at the district level was spatially heterogeneous within each country investigated throughout 2003–2013 (Figure 1; Figure 4 at http://dx.doi.org/10.5281/zenodo.44361). Spatial heterogeneity remained across all the Africa countries when adjusting for the population size of each district (Figure 5 at http://dx.doi.org/10.5281/zenodo.44361). The number of nonpolio AFP cases reported during 2003–2013 increased over time in all countries except Equatorial Guinea, although the rate of increase differed by country (Figure 6 at http://dx.doi.org/10.5281/zenodo.44361).

### Time from Paralysis Onset to Case Notification to Specimen Delivery for Laboratory Testing

The median delay between onset of paralysis and AFP case notification was <1 week in all countries across the time period analyzed, although the distribution was skewed such that 5.9% (range 2.0% [South Sudan] to 10.7% [Côte D’Ivoire]) of AFP cases were notified >3 weeks after onset of paralysis (Figure 2; Figure 7 at http://dx.doi.org/10.5281/zenodo.44361). The median delay between AFP case notification and stool sample delivery to a global polio laboratory was <1 week across 2010–2013 in Central African Republic, Kenya, Madagascar, Cote D’Ivoire, Ethiopia, and Benin, and 1–2 weeks for the remaining countries (Figure 2; Figure 8 at http://dx.doi.org/10.5281/zenodo.44361). However the distributions were also skewed such that 3.9% of stool samples were dispatched to the laboratory >3 weeks after notification.

### Spatiotemporal Model of AFP Reporting

Spatiotemporal mixed-effects modeling enabled characterization of the temporal trend and variability at the district level for each country through estimating the precision of the spatial and temporal random effects (Table 1 at http://dx.doi.org/10.5281/zenodo.44361). In all countries, there was evidence for at least 1 type of spatial random effect, indicating that the estimated district population sizes alone were not sufficient to explain differences in reporting rates. Evidence indicated overdispersion in the nonpolio reporting rate in 9 countries where a negative binomial model of nonpolio AFP case reporting provided a lower deviance information criterion value than that provided by a Poisson model. The country-specific model fits over time corresponded with the country data (Figure 6 at http://dx.doi.org/10.5281/zenodo.44361). It was possible to obtain the expected number of nonpolio AFP cases independent of time from these fitted models (Figure 1; http://dx.doi.org/10.5281/zenodo.44361). 

### Distribution of Poliomyelitis Cases and Nonpolio AFP Cases in Space and Time

Overall, compared with nonpolio AFP cases, poliomyelitis cases during the beginning of a large outbreak occurred closer together in space and time (Figure 9 at http://dx.doi.org/10.5281/zenodo.44361). In all large outbreaks during 2003–2013, most cases occurred within 100 km and 0–6 days of each other.

### Testing of Real-Time AFP Databases to Detect Polio Outbreaks

Using the Poisson space–time scan statistic to test for the presence of AFP clusters at weekly intervals resulted in prompt warnings of a polio outbreak in the 4 recent large outbreaks (Figure 3). In Tajikistan, the detection of significant clustering would have occurred on March 15, 2010, which is 39 days before official confirmation of isolation of wild poliovirus on April 23, 2010. Clustering of AFP cases would also have been detected 11 days before official confirmation of the large outbreak in Congo (October 25, 2010, and November 4, 2010, respectively). However, in Somalia, 2 large outbreaks were detected by standard surveillance methods on August 23, 2005, and May 9, 2013, respectively, whereas the clustering algorithm would not have detected significant clusters of AFP cases until September 19, 2005, and May 20, 2013, respectively. The radius of each detected outbreak was 55.4 km (Tajikistan), 239.5 km (Congo), and 10.9 km and 21.1 km (Somalia); significance levels of the alarms were all p<0.001. In other settings, the algorithm detected some smaller outbreaks of polio (Table), although the time to detection was slow (Figure 10 at http://dx.doi.org/10.5281/zenodo.44361), and other outbreaks were not detected. In all countries, with the exception of the Democratic Republic of the Congo (DRC), relatively few false alarms were raised during outbreak-free periods (Table; Figure 10 and Table 4 at http://dx.doi.org/10.5281/zenodo.44361).

**Table:** Performance of cluster detection of acute flaccid paralysis cases as an early-warning system for detection of polio outbreaks, 2003–2013*

Country	No. confirmed polio outbreaks	% Identified outbreaks	Specificity, %
Somalia	5	60	97
Tajikistan	1	100	99
Congo	1	100	96
Chad	8	62	89
CAR	4	50	91
DRC	5	80	63
Gabon	0	NA	100
Kenya	4	75	91
Mali	4	50	95
Niger	6	67	87
South Sudan	3	67	96
Madagascar	0	NA	92
Côte D’Ivoire	3	100	81
Ethiopia	5	40	93
Equatorial Guinea	0	NA	100
Cameroon	5	20	93
Benin	2	100	94
Angola	4	100	96
Ghana	1	100	92
Burkina Faso	2	0	88

*Outbreak is defined as >2 reported cases of wild poliovirus type 1 or 3 or circulating vaccine-derived poliovirus poliomyelitis <6 mo apart in the given country. CAR, Central African Republic; DRC, Democratic Republic of the Congo; NA, not applicable.

Overall, the space–time permutation scan statistic performed less well than the Poisson space–time scan statistic. The space–time permutation scan statistic would have resulted in a later detection of the 2010 Tajikistan and Congo outbreaks, and it detected fewer outbreaks in other countries (Tables 5 and 6 at http://dx.doi.org/10.5281/zenodo.44361).

## Discussion

Maintaining high-quality surveillance for polio outbreaks is essential to achieve global eradication of poliomyelitis. The longer the delay between the start of a polio outbreak and its detection (and subsequent response), the higher the chance of wide-scale spread and reestablished transmission. The large outbreak in Tajikistan in 2010 was detected relatively late (*10*), and during 2009–2010, outbreaks in Angola, Chad, DRC, and Sudan have led to reestablished transmission (*5*).

The duration of time between the onset of symptoms in the first reported polio case and confirmation of an outbreak can be prolonged due to delays in sending stool samples for laboratory testing and the time taken to perform the test. In addition, many countries do not consistently perform adequate stool collection to test for the presence for poliovirus (*22*). Our findings show that, compared with nonpolio AFP cases, poliomyelitis cases cluster in time and space, and that, in some instances, detection of spatiotemporal clustering of all-cause AFP cases can provide an early warning of outbreaks. Such a method has been shown to be an effective early-warning system for outbreaks of other infectious diseases (*23*–*25*). The method could be run on a weekly basis, as new AFP cases enter the database, and detection of a significant cluster would warrant fast-track laboratory processing of the stool samples from the associated AFP patients and alert countries to prepare for a possible outbreak.

By creating a real-time database, in which AFP cases were assumed to enter the database on the date of notification (best-case scenario of reporting), and running the spatiotemporal scan statistic at weekly intervals, an early warning of the large 2010 Tajikistan outbreak could have been raised 39 days before the date that the outbreak was officially confirmed. If outbreak response immunization campaigns had commenced 2–4 weeks earlier, substantially more poliomyelitis cases would have been prevented (*10*). In addition, an early-warning alarm of the 2010 Congo outbreak could have been raised 11 days before official confirmation. Therefore, incorporation of this early-warning system into the polio information system would benefit the Global Polio Eradication Initiative (GPEI). Although we found that the scan statistic would not have raised an early warning regarding the large 2005 and 2013 outbreaks in Somalia, the dates of the alarm were not long after the dates of official outbreak confirmation.

The algorithm performed less well at detecting much smaller outbreaks that have occurred during the past decade in countries of sub-Saharan Africa. During these outbreaks, the initial growth rate was relatively low compared with that in the outbreaks in Tajikistan, Congo, and Somalia, meaning there was little temporal clustering of polio cases. However, even if the sensitivity of early outbreak detection is not high for small outbreaks, the large outbreaks for which it does provide an early warning and hence a faster response will be of public health benefit, enabling more rapid outbreak control and a reduction in the number of poliomyelitis cases. The algorithm can be automated and, after future work to test the algorithm in other settings, would complement the current surveillance system.

Part of the polio endgame strategy is the globally synchronized removal of serotype 2 OPV from routine immunization in April 2016 (*4*). After this transition, there is a risk that cVDPV2 outbreaks will arise as population immunity against serotype 2 declines. Therefore, surveillance for cVDPV2 outbreaks will be critical during the transition period. Our results show that the algorithm we used would have generated alarms during cVDPV outbreaks in DRC, Cameroon, Kenya, and Somalia (Figure 10 at http://dx.doi.org/10.5281/zenodo.44361) and, thus, could be of help during the vaccine transition period.

A critical feature of an early-warning system is the false-alarm rate. A system with infrequent false alarms could benefit countries by providing a means to check the level of outbreak preparedness. However, a system that results in frequent false alarms is likely to be ignored when a true alarm is raised. In general, the false-alarm rate in our study was relatively low; an exception occurred in DRC, where false alarms would have been raised in late 2012–2013.

To obtain the expected proportion of AFP cases reported at the district level in the absence of a polio outbreak over a given time period, we fitted a spatiotemporal regression model to the incidence of reported nonpolio AFP during 2003–2013. We found that the number of nonpolio AFP cases reported per district was not simply a function of population size and that reporting is heterogeneous within countries. Subnational heterogeneous AFP reporting has been demonstrated at the first administrative level (province) in many settings (*6*,*26*,*27*), but fewer studies have investigated differences at the district level. Possible errors in population estimates, which could arise, in part, due to infrequent censuses, are a potential explanation for some of the heterogeneous reporting. Heterogeneities in nonpolio AFP reporting may also occur at the subnational level, reflecting differential access of populations to healthcare facilities (*28*), differences in security across the country (*26*), and local transmission of other infectious causes of AFP, such as nonpolio enteroviruses (*29*).

The number of reported nonpolio AFP cases has increased over time. We did not account for population growth in the spatiotemporal regression model of nonpolio AFP reporting, but population growth, along with improved surveillance, is likely to be a contributing factor toward the observed increase in reporting in most countries. When testing for AFP clustering in the real-time AFP database, we based the expected incidence of AFP in the absence of an outbreak only upon reported AFP cases in the preceding 2 years from the current week of surveillance. Therefore, the general time trend toward increased reporting of AFP cases would not give lead to false identification of clusters unless there was a large shift in reporting practices during those years. We assume that geographic differences in the incidence of reported AFP cases do not change over time. The low false-alarm rate in the majority of countries suggests that this is a reasonable assumption. However, the spatial random effects in our model could also be updated annually to account for such changes.

During the years of our study, no record was kept of when AFP cases were recorded in a central database. Thus, a limitation of our work is that we assumed that AFP cases were reported to the polio information system on the date the case was notified by local healthcare providers. In practice there may be further delays in collation of local information into the global polio information system AFP database. We compared the date of notification of AFP cases by local healthcare providers with the date of entry into this database by downloading this database every week during 2015 and found a median delay of 25 days (interquartile range 21–32 days) (Figure 11 at http://dx.doi.org/10.5281/zenodo.44361). If this delay were to persist it would postpone the date of an early-warning alarm by the duration of the delay; thus, there is a strong case for faster collation of data into national and global databases.

Polio outbreaks that are detected late will threaten the progress of the GPEI, and consequently there is a need to strengthen ongoing surveillance. Although future work is required to test our algorithm in other settings, we have shown that integrating an automated early-warning system based on detection of AFP clusters into the polio information system could be of value to the GPEI, helping to identify large outbreaks earlier and stop transmission faster.
